# Supplementary material for: Fluorescence microscopy of piezo1 in droplet hydrogel bilayers
Source: Channels (Austin). 2019 Mar 18;13(1):102–9. doi: 10.1080/19336950.2019.1586046 (PMC6527062; doi:10.1080/19336950.2019.1586046)
Supplement: Supplemental Material [file kchl-13-01-s0001.zip › Supplementary_Movie_Captions_20190205.docx]

**Supplementary Movie 1. Background autofluorescence in DHBs**. Autofluorescence from a DPhPC bilayer with interior consisting of empty azolectin liposomes. Exposure time: 100 ms; Laser power: 0.2 µW/um^2^; Image brightness scaled to range from 300-1600 a.u, on Andor EMCCD Camera (see Methods). Whole image is 88.68 µm by 88.68 µm. Movie is played at 10 fps (100 ms exposures) with JPEG compression.

**Supplementary Movie 2. Fluorescence of hPiezo1-GFP inserted in DHB**. hPiezo1-GFP can be observed diffusing throughout the bilayer. Exposure time: 100 ms; Laser power: 0.2 µW/um^2^; Image brightness scaled to range from 300-1600 a.u, on Andor EMCCD Camera (see Methods). Whole image is 88.68 µm by 88.68 µm. Movie is played at 10 fps (100 ms exposures) with JPEG compression.

**Supplementary Movie 3. Fluorescence of hP1-GFP in DHBs with 8% cholesterol**. hP1-GFP diffusing in a bilayer composed of 8:92 cholesterol to DPhPC. Exposure time: 100 ms; Laser power: 0.3 µW/um^2^; Image brightness scaled to range from 150-300 a.u on Hamamatsu 95B camera (see Methods). Whole image is 132 µm by 132 µm. Movie is played at 10 fps (100 ms exposures) with JPEG compression.

**Supplementary Movie 4. Fluorescence of GFP in solution above bilayer of DHBs**. Positive control for fluorescence, but not membrane protein insertion, observed using a DPhPC bilayer with an interior of GFP-filled azolectin liposomes (fluorophore present, but no membrane protein). Exposure time: 100 ms; Laser power: 0.3 µW/um^2^; Image brightness scaled to range from 150-300 a.u on Hamamatsu 95B camera (see Methods). Whole image is 132 µm by 132 µm. Movie is played at 10 fps (100 ms exposures) with JPEG compression.
